# Clinical effects of chemotherapy combined with Bevacizumab and Olaparib in treating advanced ovarian cancer and its effect on serum tumor markers and T-lymphocyte functions

**DOI:** 10.12669/pjms.41.6.10843

**Published:** 2025-06

**Authors:** Dan Liu, Ying Jiao, Lin He, Zhifeng Yang

**Affiliations:** 1 Dan Liu Department of Oncology, Baoding Second Hospital, Baoding 071000, Hebei, China; 2 Ying Jiao Department of Oncology, Baoding Second Hospital, Baoding 071000, Hebei, China; 3 Lin He Tianjin Occupational Diseases Precaution, Therapeutic Hospital, Tianjin 300011, China.; 4 Zhifeng Yang Department of Oncology, Baoding Second Hospital, Baoding 071000, Hebei, China

**Keywords:** Advanced Ovarian Cancer, Bevacizumab, Combined Chemotherapy, Olaparib

## Abstract

**Objective:**

To evaluate the clinical efficacy of chemotherapy combined with bevacizumab and olaparib in treating advanced ovarian cancer and investigate the effect of such combination treatment on serum tumor markers and T-lymphocyte function.

**Methods:**

This was a retrospective study. A total of 120 patients with advanced ovarian cancer admitted to Baoding Second Hospital and Baoding First Central Hospital from January 2022 to June 2024 were randomly divided into two groups(n= 60 each group): the control group received standard paclitaxel and carboplatin chemotherapy(TP regimen), while the study group was administered bevacizumab infusions and oral olaparib, in addition to the exact treatment provided for the control group. Comparisons were made between the two groups regarding the clinical efficacy and adverse drug reactions.

**Results:**

The overall response rate was 92% and 68% in the study group and the control group, respectively, suggesting a difference of statistical significance(*p*=0.00). Furthermore, post-treatment levels of CA153, CA125, CA199, and other markers were significantly lower in the study group than in the control group(all *p*= 0.00). Meanwhile, the post-treatment levels of CD3^+^, CD4^+^, and CD4^+^/CD8^+^ were significantly higher in the study group compared to the control group (all *p* = 0.00). Moreover, the post-treatment levels of TNF-a, CRP, IL-6, and other markers were significantly lower in the study group than in the control group (all *p*= 0.00).

**Conclusion:**

The combined use of conventional chemotherapy, bevacizumab, and olaparib demonstrates significant clinical efficacy in the treatment of advanced ovarian cancer, which can improve cellular immune function, lower tumor marker levels.

## INTRODUCTION

Ovarian cancer, a common malignant tumor of the female reproductive system, is characterized by its insidious onset and rapid progression. Due to the vague early symptoms, more than 75% of patients are already in the advanced stages of the disease at the time of diagnosis.^1^ It is challenging to completely remove the tumor lesions through surgical intervention, and the efficacy of such procedures is often suboptimal, resulting in a reduced survival rate and a significant impact on patients’ quality of life. Although ovarian cancer can be managed with treatments like radiotherapy, chemotherapy, and tumor cell reduction surgery, the persistent challenges include post-treatment tumor cell spread or metastasis, and high recurrence rates. Armstrong et al.^2^ reported that the five-years survival rate of ovarian cancer only marginally increased by 0.4% with surgery, radiotherapy, and chemotherapy, significantly lagging behind the improved survival rates achieved in the treatment of other malignant tumors. While the conventional paclitaxel-based chemotherapy is widely used for cancer treatment, it often shows limited efficacy.

In addition, some patients see minimal improvement in serum tumor markers. Cisplatin, another common chemotherapy drug for cancer treatment, frequently encounters resistance issues. While the initial response to platinum-based chemotherapy is as high as 80%, the eventual recurrence and mortality in most advanced-stage patients are attributed to acquired resistance.^3^ Therefore, patients with late-stage ovarian cancer require comprehensive treatment.^4^ Molecular-targeted therapy, a relatively new treatment approach, offers the potential to inhibit tumor growth through specific targets with minimal damage to normal tissues. Bevacizumab, a monoclonal antibody-targeted drug, exerts its anti-tumor effect by inhibiting tumor blood vessel formation. Olaparib, a poly (ADP-ribose) polymerase (PARP) inhibitor^5^, represents a novel class of targeted-therapy drugs for ovarian epithelial cancer. Olaparib has demonstrated excellent efficacy in treating advanced ovarian cancer, as well as other tumors such as ovarian cancer, fallopian tube cancer, primary peritoneal cancer, and breast cancer.

It is currently approved by the FDA for treating recurrent ovarian cancer as a standalone drug and in combination with other targeted therapies and cytotoxic drugs, especially for patients who have received at least two different platinum-based chemotherapy regimens.^6^ This study aimed to investigate the clinical efficacy and safety of a combined intervention approach involving bevacizumab, olaparib, and conventional chemotherapy for non-surgical patients with advanced ovarian cancer.

## METHODS

This was a retrospective study. One hundred and twenty patients with advanced ovarian cancer admitted to Baoding Second Hospital and Baoding First Central Hospital from January 2022 to June 2024 were randomly divided into control group (n= 60) and study group (n=60) according to different treatment methods. The study group was aged 33– 60, with an average age of 51.46±5.35, while the control group had an age range of 32–58, with an average age of 51.28±5.27. All patients had a KPS of ≥70 and were physically suitable for participation in this study. No significant differences were observed in the general data between the two groups, indicating comparability (Table-I).

**Table-I:** Comparative analysis of general data between the two groups (x̄ ± S) n = 60.

	Study Group	Control Group	t/χ^2^	p
Age (years)	51.46±5.35	51.28±5.27	0.19	0.85
Weight (kg)	56.74±6.82	56.59±6.65	0.12	0.90
BMI (kg/m^2^)	20.31±2.17	19.97±2.04	0.88	0.37
KPS	83.72±7.95	83.43±6.91	0.22	0.83
Tumor Stage			0.43	0.51
Stage III(n%)	48(80%)	45(75%)		
Stage IV(n%)	12(20%)	15(25%)		
***Pathology***				
Serous carcinoma (n%)	31(52%)	30(50%)	0.03	0.86
Mucinous carcinoma (n%)	16(27%)	18(30%)	0.16	0.68
Endometrial carcinoma (n%)	8(13%)	10(17%)	0.26	0.61
Undifferentiated carcinoma (n%)	5(8%)	2(3%)	1.36	0.24

p>0.05.

### Ethics approval:

The study was approved by the Institutional Ethics Committee of Baoding Second Hospital (No.: 2023[086]; Date: December 1, 2023), and written informed consent was obtained from all participants.

### Inclusion criteria:

- Diagnosed with ovarian cancer through imaging examination and confirmed by pathological diagnosis according to the diagnostic criteria for advanced ovarian cancer(stage III–IV).^7^
- Karnofsky Performance Score(KPS) > 70, and expected survival time > 3 months.^8^
- 30–60 years old.
- No history of treatment for the condition.
- Complete clinical data.
- Absence of obvious neurological and cognitive impairment, and capability of participating in the study.
- Agreement to this study protocol with signed the consent form.

### Exclusion criteria:

- Any mental diseases preventing the patient from cooperation with the study.
- Immune dysfunction or severe infection.
- Liver and kidney dysfunction.
- Allergies to the drugs involved in the study.
- Malignant tumors in other organs.
- history of chemoradiotherapy and other;
- Incomplete clinical data.

The control group received the conventional TP chemotherapy as follows: Intravenous (IV) infusion of paclitaxel at 175 mg/m^2^ on day one of each treatment cycle, with carboplatin at an AUC of five administered on day two of each treatment cycle. Each cycle lasted for three weeks, and the treatment consisted of six cycles. Meanwhile, symptomatic treatments such as antiemetics, gastrointestinal mucosal protection, and white blood cell elevation were given during the treatment.

The study group received a bevacizumab injection at 7.5 mg/kg dissolved in 0.9% sodium chloride solution via IV infusion, in addition to the exact treatment provided for the control group. The first infusion was controlled to take place within 1.5 hours, with subsequent infusions controlled to occur within 0.5–1.0 hours. Specifically, the infusion was completed one hour before the initiation of chemotherapy, three times a week. Olaparib was administered orally at 200 mg twice daily. The treatment continued until disease progression or occurrence of unacceptable toxicity.

### Outcome measures:

*Clinical Efficacy Evaluation:* Both groups of patients underwent contrast-enhanced CT or MRI examination every month after treatment. Disease control was determined based on imaging results, which can be categorized as follows: Complete Response (CR): Complete disappearance of tumor, lasting > 1 month; Partial Response (PR): Tumor reduction ≥ 50%, lasting > one month; Stable Disease (SD): Tumor reduction ≥ 50% or increase ≤ 25%, lasting > one month; Progressive Disease (PD): Tumor growth. Overall Response Rate (ORR) = (CR + PR) cases / total cases × 100%; Disease Control Rate (DCR) = (CR + PR + SD) cases / total cases × 100%;^9^

*Tumor Marker Levels:* Peripheral venous blood (5 ml) was collected from each patient before and at one month after treatment to detect tumor markers such as CA153, CA125, and CA199.

*T-Lymphocyte Subset Analysis:* Peripheral venous blood was collected from patients before treatment and after treatment to detect T-lymphocyte subsets CD3^+^, CD4^+^, CD8^+^, and CD4^+^/CD8^+^. Changes in these subsets between the two groups were compared and analyzed.

*Comparison of Inflammatory Factor Levels:* Peripheral venous blood (5 ml) was collected from each patient before treatment and the morning after treatment. Inflammatory factors such as tumor necrosis factor-alpha (TNF-α), C-reactive protein (CRP), and interleukin-6 (IL-6) were measured through enzyme-linked immunosorbent assay (ELISA).

*Assessment of Adverse Drug Reactions:* Adverse drug reactions occurring within one month after medication were recorded for both groups, including nausea, vomiting, bone marrow suppression, diarrhea, liver damage, and cardiotoxicity. The maximum follow-up time for patients in both groups was six months, and case data collection ceased in December 2023.

***Statistical analysis:*** All data were statistically analyzed using SPSS 20.0 software. Measurement data were expressed as “mean ± standard deviation *(x̄ ± S)*”. Intergroup data analysis was conducted using independent sample *t*-tests. Paired *t*-tests and chi-squared tests were used for intragroup data analysis and rate comparison, respectively. A *p*-value of < 0.05 was considered statistically significant.

## RESULTS

A comparison of pre- and post-treatment clinical efficacy between the two groups (Table-II) indicated that the ORR of the study group was 92%, while the control group showed an ORR of 68%. The study group exhibited a significantly higher ORR compared to the control group (*p* = 0.00). The changes in pre- and post-treatment serum tumor markers within both groups are shown in Table-III. Before treatment, there were no significant differences in CA153, CA125, CA199, and other markers between the two groups (all *p* > 0.05).

**Table-II:** Comparison of Pre- and Post-Treatment clinical efficacy between the two groups (x̄ ± S) n = 60.

Measures	CR	PR	SD	PD	ORR
Study Group	19	26	10	5	55(92%)
Control Group	13	21	15	11	41(68%)
c^2^					9.09
*p*					0.00

p < 0.05.

**Table-III:** Comparison of Pre- and Post-Treatment serum tumor markers between the two groups (x̄ ± S) n = 60.

Measures	Observation Point	Study Group	Control Group	t	p
CA153 (U/ml)	Pre-treatment	50.36±5.64	50.48±5.17	0.12	0.90
	Post-treatment*	23.77±2.12	25.49±2.20	4.36	0.00
CA125 (U/ml)	Pre-treatment	124.37±11.25	123.94±12.07	0.48	0.64
	Post-treatment*	41.25±6.08	44.30±6.14	2.74	0.00
CA199 (U/ml)	Pre-treatment	103.18±13.07	102.88±12.64	0.44	0.66
	Post-treatment*	40.32±7.94	44.05±7.50	2.91	0.00

* p < 0.05.

However, after treatment, the study group exhibited significantly lower levels of the aforementioned markers compared to the control group (all *p* = 0.00). No significant differences were observed in the pre-treatment levels of CD3^+^, CD4^+^, CD8^+^, and CD4^+^/CD8^+^ between the two groups (all *p* > 0.05). In contrast, after treatment, the study group demonstrated significantly higher levels of CD3^+^, CD4^+^, and CD4^+^/CD8^+^ than the control group (all *p* = 0.00). Notably, changes in CD8^+^ were not significant (*p* = 0.21) (Table-IV).

**Table-IV:** Comparison of Pre- and Post-Treatment changes of t-lymphocyte subsets within the two groups (x̄ ±S)n=60.

Measures		Study Group	Control Group	t	p
CD3^+^(%)	Pre-treatment	43.93±7.82	44.05±7.42	0.09	0.73
	Post-treatment*	49.55±7.06	46.07±7.10	2.70	0.00
CD4^+^(%)	Pre-treatment	28.60±7.15	27.89±7.06	0.55	0.58
	Post-treatment*	34.58±5.24	31.40±5.72	3.18	0.00
CD8^+^(%)	Pre-treatment	23.68±3.45	23.17±3.09	0.85	0.40
	Post-treatment	25.72±3.75	24.90±3.41	1.25	0.21
CD4^+^/CD8^+^	Pre-treatment	1.16±0.12	1.20±0.18	1.43	0.15
	Post-treatment*	1.70±0.27	1.47±0.03	6.56	0.00

* p<0.05.

There was no significant difference in TNF-a, CRP, IL-6, and other inflammatory factors before treatment between the study and control groups (p > 0.05). After treatment, measures such as TNF-a, CRP, and IL-6 in the study group were significantly lower than those in the control group, with statistically significant differences (p = 0.00) (Table-V). The incidence of adverse drug reactions was 38% in the study group and 28% in the control group, representing no significant difference between the two groups (*p* = 0.28) (Table-VI).

**Table-V:** Comparison of Pre- and Post-Treatment changes of inflammatory factors within the two groups (x̄ ± S) n = 60.

Measures		Study Group	Control Group	t	p
CRP (mg/L)	Pre-treatment	38.74±6.85	38.89±6.57	0.12	0.90
	Post-treatment*	23.40±4.77	26.53±5.27	3.68	0.00
IL-6 (ng/L)	Pre-treatment	11.81±3.36	11.43±3.25	0.63	0.52
	Post-treatment*	6.59±2.37	9.64±3.10	6.05	0.00
TNF-ɑ (ng/L)	Pre-treatment	27.63±6.72	28.05±6.08	0.36	0.71
	Post-treatment*	13.47±3.52	16.21±3.18	4.47	0.00

* p < 0.05.

**Table-VI:** Comparison of Post-Treatment adverse drug reactions between the two groups (x̄ ± S) n = 60.

Group	Nausea and Vomiting	Bone marrow suppression	Diarrhea	Liver damage	Arthralgia	Cardiotoxicity	Incidence
Study	2	5	3	2	3	2	23(38%)
Control	2	4	2	3	2	2	17(28%)
c^2^							1.19
*P*							0.28

## DISCUSSION

This study shows that the TP chemotherapy combined with bevacizumab and olaparib has an ORR of 92% in patients with advanced ovarian cancer, significantly higher than the 68% ORR in the control group(*p*= 0.00). After treatment, the levels of CA153, CA125, CA199, and other markers in the study group were significantly lower than those in the control group (all *p* = 0.00). The analysis suggests that bevacizumab, as a targeted therapy, exerts anti-VEGF effects by inhibiting the formation of new tumor blood vessels, reducing their permeability, neutralizing the bioactivity of VEGF, and stabilizing the tumor vascular system. When combined with olaparib, it demonstrates a synergistic effect, effectively improving the anti-cancer effect of chemotherapy drugs, promoting lesion clearance, inhibiting neovascularization, and reducing tumor cell recurrence and metastasis. In the context of ovarian cancer, common tumor markers, such as CA153, CA125, and CA199, are often secreted into the bloodstream in significant quantities due to stimulation by tumor cells, leading to an abnormal increase.^10^

Elevated levels of such common inflammatory factors as TNF-a, CRP, and IL-6 indicate increased tumor activity and higher malignancy, which can exacerbate the patient’s condition. The findings of this study show that after six treatment cycles, the levels of TNF-a, CRP, IL-6, and other markers were significantly reduced in the study group compared to the control group (all *p*=0.00). This suggests that the combination of the TP chemotherapy regimen with bevacizumab and olaparib can alleviate the inflammatory response caused by advanced ovarian cancer and reduce tumor marker levels. Further analysis suggests that bevacizumab and olaparib can effectively inhibit inflammatory factors, limit the generation of ovarian reactive oxygen species, and eliminate free radicals in the body, thereby promoting the reduction of CA153, CA125, and CA199 and controlling disease progression.

The occurrence and progression of malignant tumors are closely related to immune function, particularly cellular immune function, which involves immune responses primarily mediated by CD3^+^, CD4^+^, and CD8^+^ cells.^11^ The present study revealed that after treatment, the levels of CD3^+^, CD4^+^, and CD4^+^/CD8^+^ were higher in the study group compared to the control group (all *p* = 0.00). The incidence of adverse drug reactions in the study group and the control group was 38% and 28%, respectively, suggesting no statistically significant difference (*p* = 0.28). This may be attributed to the use of the PARP inhibitor olaparib, which eliminates cancer cells by inhibiting gene homologous recombination defects. When combined with the TP chemotherapy, it can yield a synergistic effect, maximizing the inhibition of tumor progression, and further reducing the size of solid tumors. Additionally, olaparib possesses strong inhibitory properties and high specificity, facilitating its binding to therapeutic targets and resulting in fewer adverse reactions.

Furthermore, normal cell tissues have multiple signal pathways for repairing DNA breaks, and this inhibitor has relatively less effect on eliminating normal cell tissues, which can stimulate and alleviate immune function damage without increasing drug adverse reactions. Specifically, olaparib-based targeted therapy may enhance the body’s immune response against cancer cells by affecting immune system activity, thereby bolstering the body’s resistance to tumor progression. Consistent with our findings, Poveda et al.^12^ reached a similar conclusion that olaparib-based targeted therapy can enhance the body’s resistance to tumor progression by affecting the activity of the cellular immune system, regulating T cell function, and strengthening the body’s immune response against cancer cells.

Studies have demonstrated that ovarian cancer ranks second in terms of incidence among female reproductive tract tumors, with a five-years survival rate of only 30%.^13^ The combination of paclitaxel and carboplatin, known as the TP chemotherapy regimen, is commonly used in the treatment of ovarian cancer. On one hand, paclitaxel is a naturally occurring anticancer drug that disrupts tubulin and tubulin dimers upon administration, leading to tubulin polymerization and microtubule assembly, which, in turn, stabilizes microtubules and inhibits mitosis in tumor cells, resulting in a potent anticancer effect.^14^ Paclitaxel is used for the treatment of various cancer types, including adenocarcinoma, with lung cancer, breast cancer, and colorectal cancer being its most common applications. On the other hand, carboplatin, a second-generation platinum compound, is a broad-spectrum antitumor drug used for the clinical treatment of solid tumors such as small cell lung cancer, ovarian cancer, testicular tumors, head and neck cancer, and malignant lymphoma. It has strong cytotoxic effects and inhibits DNA synthesis in tumor cells, induces tumor cell apoptosis, reduces tumor proliferation capacity, and impedes tumor growth.^15^ The combination of these two drugs enhances the anti-tumor effect.

However, considering drug resistance induced by secondary gene mutations in tumor cells and other factors, this conventional chemotherapy regimen exhibits limited efficacy in cases of recurrent or advanced ovarian cancer.^16^ In light of the above circumstances, there is increasing use of targeted drugs in the clinical treatment of ovarian cancer, making a new trend in the management of advanced ovarian cancer. Bevacizumab, a widely used anti-tumor targeted drug in clinical practice, is a recombinant monoclonal antibody derived from vascular endothelial growth factor (VEGF). This anti-tumor agent competitively binds to VEGF, inhibiting surface receptor binding, thereby reducing VEGF activity and blocking neovascularization.

Consequently, it exerts inhibitory effects on tumor invasion and metastasis.^17^ VEGF functions as the basis for tumor cell tissue growth and metastasis, and elevated levels of VEGF in neovascularization are associated with a poorer prognosis in patients with ovarian cancer. Therefore, effective inhibition of VEGF levels can enhance anti-tumor effects.^18^ Garcia et al.^19^ suggested that bevacizumab can inhibit VEGF signaling, disrupt tumor blood vessels (both mature and leaky), normalize tumor blood vessels, decrease drug resistance, and improve drug utilization. In comparison, olaparib is a PARP inhibitor that further blocks cellular base excision repair function in cancer cells with DNA damage, leading to the inhibition of cell single-strand replication and increased DNA double-strand breaks. As a result, defects in self-repair mechanisms prevent the completion of DNA double-strand break repair, ultimately inducing apoptosis in cancer cells. In light of this, the combination of the TP chemotherapy regimen with olaparib has shown promise in improving short-term outcomes, reducing the risk of disease progression or death, with a 61% reduction in the high-risk group and an impressive 85% reduction in the low-risk group.^20^

### Limitations:

It includes a small sample size and a short follow-up period. Consequently, the survival time and long-term therapeutic effects of this treatment regimen have not been included in the study. Additionally, with the continuous introduction of new targeted drugs, there is still a lack of comparative studies between other drugs and this treatment regimen. In further clinical work, we intend to increase the sample size, extend the follow-up time, and incorporate other drug combination regimens into the research to comprehensively and objectively evaluate the long-term effects of this treatment regimen on patients.

## CONCLUSIONS

The combination of chemotherapy with bevacizumab and olaparib in the treatment of advanced ovarian cancer has demonstrated significant clinical efficacy. This combination treatment can effectively reduce the level of serum tumor markers, alleviate inflammatory reactions, and improve T-lymphocyte functions without significantly increasing adverse reactions, making it a safe and effective treatment option.

### Authors’ Contributions:

**DL** and **ZY:** Carried out the studies, participated in collecting data, and drafted the manuscript, and are responsible and accountable for the accuracy or integrity of the work.

**YJ:** Participated in its design. Performed the statistical analysis **LH:** Reviewed Literature, data collection and analysis.

All authors have read and approved the final manuscript.
